# The effects of pregabalin, solifenacin and their combination therapy on ureteral double-J stent-related symptoms: A randomized controlled clinical trial

**DOI:** 10.1590/S1677-5538.IBJU.2020.0848

**Published:** 2021-01-10

**Authors:** Siavash Falahatkar, Mohammadreza Beigzadeh, Gholamreza Mokhtari, Samaneh Esmaeili, Ehsan Kazemnezhad, Atiyeh Amin, Nadia Rastjou Herfeh, Reza Falahatkar

**Affiliations:** 1 Guilan University of Medical Sciences School of Medicine Razi Hospital Rasht Iran Urology Research Center, Razi Hospital, School of Medicine, Guilan University of Medical Sciences, Rasht, Iran

**Keywords:** Pregabalin, Solifenacin Succinate, Lithotripsy

## Abstract

**Background:**

Many medical therapies have been tested to deal with urinary stent-related symptoms (USRS). Several preventive and pharmaceutical methods have been already used for better compatibility of stents. However, the existing evidence for pharmacological treatment is still controversial. This study aims to evaluate the effects of pregabalin, solifenacin, and combination therapy on ureteral double-J stent-related symptoms following ureteroscopy and transureteral lithotripsy (TUL).

**Materials and methods:**

In a randomized controlled clinical trial, from November 2017 to March 2019, 256 patients who underwent ureteroscopy were enrolled. Patients were randomly divided into four groups including: group A received pregabalin 75mg BID (twice daily), group B received solifenacin 5mg orally once daily, group C received combination of pregabalin and solifenacin and the group D (control) given no drugs.

**Results:**

One hundred and fifty-one (58.9%) males and 101 (41.1%) females were enrolled in this study with a mean age of 43.47±7 (p=0.32, p=0.67). USSQ domains score such as urinary symptoms, pain, general condition, work performance, sexual matters and additional problems were significantly differenced during second and fourth week of follow-up among study groups (p <0.0001).

In Tukey's multiple comparison test, urinary symptoms (p=0.735), pain (p=0.954) and sexual matters (p=0.080) in second week and work performance in forth week in group B was not significantly better than group D. Only group C in all indexes of USSQ showed significantly beneficial effects over group D (p <0.0001).

**Conclusion:**

Combination therapy of pregabalin and solifenacin has a significant effect on stent-related symptoms and is preferred over monotherapy of the respected medications.

## INTRODUCTION

Double-J ureteral stent has been introduced as a urological instrument for use in a broad range of urinary tract system disorders ranging from obstructive pyelonephritis, ureteral edema, ureteral perforation and kidney transplantation (1). On the other hand, this useful device highly affects the patient's quality of life. Infection, lower urinary tract symptoms (LUTS), dysuria, frequency, urgency, incomplete emptying, and sexual disorders are some of these ureteral stent-related symptoms (USRS) that affect patient's quality of life following ureteroscopy and transureteral lithotripsy (TUL) (2–4).

The exact pathophysiology of USRS remains so far unknown. There is a study showing that local irritation could causes pain and LUTS due to bladder and ureter spasm (5). Some recent studies suggest oral medications following ureteroscopy can resolve stent-related symptoms including pain, frequency, quality of life, general health, and total Ureteral Stent Symptom Questionnaire (USSQ) score (1, 6).

Pregabalin as gamma-aminobutyric acid (GABA) agent is approved by the FDA for diabetic neuropathy, central pain, headaches, and other chronic pains. Recently, some studies have shown positive effects of pregabalin on LUTS disorders (7, 8). Solifenacin as an antimuscarinic agent blocks the muscarinic receptor and is approved for overactive bladder. Recent studies have shown improvement in USRS and suggest its use for treatment of symptoms following ureteroscopy and lithotripsy (9, 10).

Following ureteroscopy and TUL, today, DJ stents are routinely used to prevent obstruction, dilate the ureter, and urine ejection, which speeds up tissue healing (11). As the pharmacological treatment of USRS is still controversial, therefore, it is so important to find a solution to decrease the symptoms.

Due to limited number of patients, a different method of scoring systems in recent studies, and short-term follow-up after discharge (11, 12), we aimed to evaluate the effects of pregabalin, solifenacin and combination therapy on ureteral double-J stent-related symptoms following ureteroscopy and TUL in patients with ureteral stone.

## MATERIALS AND METHODS

### Study Population and Design

After being approved by the Ethics Committee of Guilan University of Medical Sciences, and registration in Iranian Registry of Clinical Trials (IRCT) with reference number “IRCT20090427001853N16”, 256 patients from November 2017 to March 2019 were enrolled to the study. The study was conducted in the Department of Urology, Razi Hospital of Guilan University of Medical Sciences and informed consent was obtained from all the patients before enrollment.

Inclusion criteria were patients aged between 18 and 60 years, undergoing ureteroscopic stone extraction and needed DJ stent insertion. Patients with a history of infravesical obstruction, neurogenic or hyperactive bladder, benign prostatic hyperplasia (BPH), bilateral stent, recurrent urinary tract infection or chronic pelvic pain, hepatic failure, renal failure (GFR <30), taking chronic medications such as alpha-blockers or analgesics, bilateral ureteral obstruction or stones, past history of ureteral DJ stent, patients with pregabalin contraindications (such as: hypersensitivity, pregnancy, lactation, renal or hepatic impairment), and those undergoing other urological surgery simultaneously with urethroscopy were excluded from the study.

Based on a power of 80% and confidence interval of 95% and using the results of the study of Rageb et al. (1), a sample size of 64 patients in each group was needed for comparison of USSQ scores.

**Figure 1:**
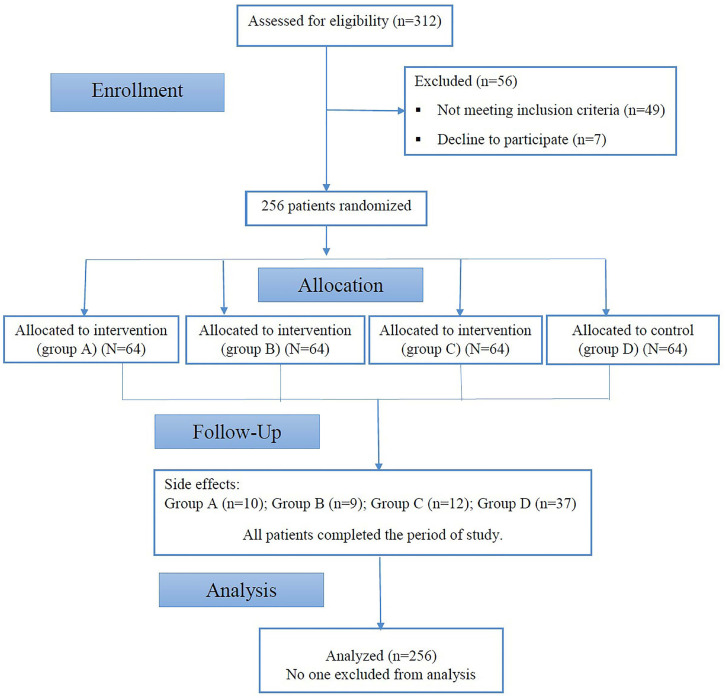
Study flow diagram.

Patients were randomly divided into 4 groups: group A (n=64) received pregabalin 75mg capsules BID, group B (n=64) received solifenacin 5mg tablets orally once daily, group C (n=64) received both pregabalin 75mg capsules BID and solifenacin 5mg tablets orally once daily, and no medication was given to those in group D as control group (n=64) (Figure-1).

### Patients assessment and outcome measurements

Laboratory tests such as urine analysis (U/A), serum creatinine, blood urea nitrogen (BUN), complete blood count (CBC), aspartate aminotransferase (AST), alanine aminotransferase (ALT), alkaline phosphatase (ALP), serum bilirubin, and serum electrolytes were obtained from all the patients.

Antibiotic prophylaxis consisted of preoperative injections of cefazolin and amikacin, and postoperative oral ciprofloxacin 500mg tablet orally BID for three days and then at a dose of 250mg tablet orally up to the end of the period. Postoperative paracetamol was given as a single dose, if needed and after discharge acetaminophen 500mg orally (up to 7 days) or diclofenac suppository 50mg was prescribed.

All procedures were performed by one expert urologist. All the procedures were performed by using Pneumatic lithoclast. The caliber and length of the DJ were adjusted to each patient and stents were inserted over the guidewire in a classic method. KUB X-ray or fluoroscopy was used to detect the proper stent location.

Frequency, nocturia, urgency, incomplete emptying, dysuria, hematuria and pain were evaluated in 2 and 4 weeks after the discharge from hospital.

“Total urinary symptoms score,” “total pain score,” “total general condition score,” “total work performance score,” “total sexual matter score,” and “additional problems score” were evaluated as total score indexes.

Persian version of Ureteral Symptom Score Questionnaire (USSQ) was used for recording the data (Appendix-1). Its reliability and validity in Iran were proved by Jabbari et al. in 2015 (13).

Stone free rate was evaluated 4 weeks after removal of the stent by ultrasonography or KUB X-ray.

### Statistical Analysis

The Total indexes and different variables of USSQ in the present study were reported as mean±SD and were statistically analyzed by SPSS software (version 16). To compare parametric factors, students T-test and chi-square test, one-way repeated-measures ANOVA were used. Tukey's multiple comparison test was used to compare the difference between each pair of means in groups and Dunnett's Test were used for comparing control to treatment groups. A p-value less than 0.05 (p ≤0.05) was considered statistically significant.

## RESULTS

Two hundred and fifty-six patients including 151 males and 101 females in this study, were randomly enrolled into four groups (each group of 64 patients). The mean age was 43.47±7.6 years old. On the base of sex and age there were no significant differences among the groups (p=0.32, p=0.67).

### Urinary tract symptoms

On the other hand, urgency at 2 weeks and 4 weeks follow-up periods was lower in the group D. During follow-up periods in group C, frequency, nocturia, hematuria, pain, and analgesic use were lower but in the group D urgency was lower (Table-1).

**Table 1:** Comparisons of efficacy outcomes (USSQ total score and its domains) among our study groups.

Variables		Groups				
		A	B	C	D	P
Frequency	2^nd^ week	0.47±3.33	041±2.8	0.5±2.59	1.04±3.41	0.0001
	4^th^ weeks	0.48±2.33	1.2±0.41	1.19±0.4	1.41±3.03	0.0001
Nocturia	2^nd^ week	0.48±3.33	3	0.81±2.39	0.64±3	0.0001
	4^th^ weeks	2	2	0.5±1.59	0.75±2.81	0.001
Urgency	2^nd^ week	0.88±3.89	0.49±3.61	2.81±0.75	1.8±0.76	0.0001
	4^th^ weeks	2.33±0.47	2.61±0.49	2.06±0.89	1.61±0.81	0.0001
Incomplete Emptying	2^nd^ week	2	2	2	1.98±0.9	0.1
	4^th^ weeks	2	1	1	1.8±0.77	0.0001
Dysuria	2^nd^ week	3.22±0.42	2.39±0.49	0.38±2.83	2.59±1.04	0.0001
	4^th^ weeks	2	0.49±1.39	1.83±0.38	2.59±1.03	0.0001
Hematuria	2^nd^ week	2	1.25±0.44	1	2.59±1.38	0.0001
	4^th^ weeks	1	1	1	2.41±1.38	0.0001
Pain	2^nd^ week	2.84±0.37	3.62±0.49	1.83±0.77	3.41±1.01	0.0001
	4^th^ weeks	2	2.62±0.49	1.98±0.65	3.41±1.01	0.0001
Analgesic Use	2^nd^ week	2	3	1.64±0.84	2.84±1.31	0.0001
	4^th^ weeks	1	2.62±1.4	1	1	0.0001
Antibiotic Use	2^nd^ week	1	1	1	1.34±0.48	0.0001
	4^th^ weeks	1	1	1	1.38±0.49	0.0001
USSQ scores						
Urinary Symptoms	2^nd^ week	31.06±5.08	28.64±1.85	26.39±3.21	29.3±3.6	0.0001
	4^th^ weeks	21.81±1.58	19.25±1.49	16.75±2.31	20.66±4.49	0.0001
Pain	2^nd^ week	21.88±1.86	28.98±2.62	17.48±2.58	29.14±3.89	0.0001
	4^th^ weeks	12.47±1.08	19.66±1.49	12.97±1.21	20.72±3.68	0.0001
General Condition	2^nd^ week	15±1.01	17.61±1.87	15.31±2.02	18.9±2.17	0.0001
	4^th^ weeks	11.77±0.64	13.48±1.36	12.97±1.21	12.39±2.02	0.0001
Work Performance	2^nd^ week	19.9±1.9	19.11±0.9	16.9±1.56	22.56±3.18	0.0001
	4^th^ weeks	15.05±1.9	15.56±1.11	13.03±1.46	15.95±3.39	0.0001
Sexual Matters	2^nd^ week	6.08±0.86	8.08±1.35	5. 92±0.95	7.59±1.05	0.0001
	4^th^ weeks	5.42±0.5	5.86±0.85	5.12±0.33	6.77±0.83	0.0001
Additional Problems	2^nd^ week	7.58±0.56	11.16±0.76	8.53±1.04	9.16±1.6	0.0001
	4^th^ weeks	6.58±0.69	8.61±0.49	6.72±0.86	8.09±1.27	0.0001

Among all groups incomplete emptying was not significantly different in follow-up period (p=0.1).

The result has shown that dysuria during the 4-week follow-up period was lower in group B than other groups and the reduction of this symptom was statistically significant (p <0.0001). There was no significant difference between all groups on the base of antibiotics using in follow-up periods.

### Total indexes of USSQ

In group B, total urinary symptom score and total pain score were lower during follow-up periods.

In group C, total work performance score and total sexual matter score were lower during follow-up periods.

We observed lower total pain score, total general condition score, and “total additional problems score at 4 weeks after admission in the group A (Table-1).

Multiple comparison showed that group A and group C were significantly superior to group D (control), in all indexes and variables of USSQ and in both the second and fourth weeks of treatment. But group B regarding urinary symptoms score (p=0.607) and pain score (p=0.916) in second week and also work performance score (p=0.606) in forth week was not better than group D.

In multiple comparison, group B regarding urinary symptoms (p=0.735), pain (p=0.954) and sexual matters score (p=0.080) in second week and work performance score in forth week was not significantly better than group D. Only group C (pregabalin+solifinacin) in all indexes of USSQ showed significantly beneficial effects over control group (D) (Appendix-2).

### Side effects

The reported drug-related side effects were mild, tolerable and not life threatening in all the study groups and no patients discontinued the medication treatments because of that. Flushing was not reported in the study group which received Solifenacin (group B). But it was encountered in two and one case in group A and C versus eleven cases in group D. Dry mouth was reported in 23 patients (1, 3, 7 and 12 patients in groups A, B, C and D, respectively). Also, 33 of our patient complained of drowsiness (4 cases in group A, 7 case in group B, 5 cases in group C and 17 case in group D), while 17 cases had dizziness (3 cases in group A and 2 case in group B, 4 cases in group C and 8 case in group D). There was no report of body pain in the group which received both drugs (group C) and just 2, 1 and 4 patients had body pain in groups A, B and D, respectively. Headache was recorded in one patient in group A, one in group C and three patients in group D.

## DISCUSSION

There are many different procedures to treatment ureteral stones. Adjunctive medical expulsive therapy (MET) with tamsulosin had an important role to pass ureteral stones (14).

TUL, ESWL, laparoscopic surgery, PCNL are other options to treatment ureteral stones. A meta-analysis demonstrated that LU and PCNL are good procedures for patients with large proximal ureteral stones (15).

Percutaneous nephrolithotomy (PCNL) is recommended as the first-line treatment of choice for renal stones more than 2cm in diameter and retrograde flexible ureteroscopy (FURS) is an effective and safe alternative to PCNL and in treating intermediate-size renal stones (2-3cm). Following these procedures sometimes it is mandatory to insert DJ (16, 17).

DJ stent is known as useful and routine instrumentation in urology surgeries that reduces obstruction of the upper urinary tract, deflects the urine outflow, helps ureter dilatation for stone passing and helps to heal the tissue by reduction of inflammation. However, it can cause symptoms and even giant stone formation related to a stent that affects more than 80 percent of the patients (2, 11, 18).

Several stent-related symptoms have been reported in recent studies such as frequency, hematuria, nocturia, and pain that are inevitable after DJ stent insertion. Incomplete emptying, frequency, urgency, dysuria, pain and hematuria are the most common symptoms reported in some studies (12, 19). The pathophysiology of USRS is unclear, but some study suggested perhaps local irritation, urine reflux, bladder spasms, and inflammation could play a role (12).

There are different ideas about the management of USRS, the first is the management with the improvement of materials and structure of the stent to reduce stent-related symptoms and the second is to control postoperative symptoms by using various drugs. GABA agents such as pregabalin and antimuscarinic agents such as Solifenacin are some of these drugs that are used alone or in a combination to relieve USRS after stent insertion (1, 20–22).

Despite positive effects of alpha-blockers or antimuscarinic agents alone or in combination therapy in reducing stent-related symptoms, Park et al. showed no significant difference on stent-related symptoms based on USSQ scores between groups that used alpha-blockers or anticholinergic agents single or in combination treatment (21, 23).

The study of Joshi et al. was the first study that introduced USSQ questionnaire as sensitive and essential criteria for comparing different drugs to evaluate the sensitivity and effectiveness of them in patients following DJ insertion (2, 11).

The present study enrolled 256 patients underwent ureteroscopy and TUL, 152 males and 104 females, the mean age in the present study was 43.52±7.75. The mean age in the study of Ragab et al. was lower in comparison with the present study (1). Four weeks after admission, frequency (0.48±2.33), total pain (12.47±1.08), general condition (11.77±0.64) sexual matters scores (5.42±0.5), and additional problems (0.69±6.58), all were lower in the group A than group B (1.2±0.41; 19.66±1.49; 13.48±1.36, 5.86±0.85 and 8.61±0.49, respectively), group C (1.19±0.4; 12.97±1.21; 12.97±1.21, 5.12±0.33 and 6.72±0.86, respectively) and group D (1.41±3.03; 20.72±3.68; 12.39±2.02, 0.83±6.77 and 1.27±8.09, respectively). On the other, after 4 weeks of treatment, incomplete emptying, nocturia, dysuria and urinary symptoms in group A were higher than other groups. Group B had lowest dysuria (0.49±1.39).

While analgesic use, after 4 weeks, decreased in all 4 groups, this decrease was much lower in group B (2.62±1.4) than the other groups (all were 1).

At the end of the 4-week period, group C had better results in nocturia (0.5±1.59), pain (1.98±0.65), total urinary symptoms score (16.75±2.31) and total work performance score (13.03±1.46) compared to other groups. Ragab et al. (1) demonstrated an improvement in total USSQ score in patients who received combination therapy of pregabalin and solifenacin that was also similar to our results. They did not report urinary symptoms in detail, but in their study frequency, nocturia, urgency, dysuria, hematuria were lower in group that received solifenacin than group received pregabalin (1). El-Nahas et al. demonstrated a higher improvement rate in USSQ score in patients received solifenacin than group received tamsulosin (22).

The pain score was lower in the group that received pregabalin (group A) in 2 weeks and four weeks period after admission, which is same the result of a study by Ragab et al. (1).

The overall results of the USSQ in our study showed that the scores of urinary symptoms, pain, general health, professional performance and sexual desire after 2 weeks were higher than the result of a study by Ragab et al. (1), which was performed in the period of 2 weeks after the stent insertion, however, they were lower than the mean scores of their study after 4 weeks, which indicates that long-term follow-up of patients after stent placement is required in studies.

In this study, after 4 weeks of treatment, only the total score of USSQ in group A compared to group D was statistically significant. Also, the difference in urgency in second and fourth week and analgesic use in fourth week in group B was significant.

In group C, the differences in frequency, urgency, nocturia, dysuria, pain and urinary symptoms in second and fourth week after stent insertion and analgesic use in second week were significantly higher than group D. In one study the total score of USSQ, general health and quality of life in group receiving pregabalin and solifenacin significantly changed better than each other groups, separately (1).

Although the result of a study by El-Nahas et al. (22) was similar to our study, anticholinergic and α-blockers users showed significant improvement compared to control group, in another study the authors concluded that there were no significant effect in stent-related symptoms of those who received anticholinergic and α-blockers or their combination (23).

In the present study, there was a significant effectiveness in “frequency”, “nocturia”, “urgency”, “incomplete emptying”, “hematuria”, “pain”, “analgesic use”, “total urinary symptom score”, “total pain score”, “total general condition score”, “total work performance”, and “total sexual matters score” in the group C than other groups. “Incomplete emptying” and “antibiotic use” were equal in the groups.

In the present study, the side effects of drug were lower in the group A. No patient reported flushing in the group B. Flushing and body pain were higher in group A than other treatment groups. In contrast, dry mouth and drowsiness were lower in the group A rather than other groups.

There was no complaining of body pain in group C and also, headache and flushing were recorded just in one patient in this group. Most of the side effects were minor and self-limited in study groups. The safety, efficacy, and tolerability of pregabalin and solifenacin have been approved in previous clinical studies (1, 6, 9). The reported side effects were surprisingly more intense in control group but all were tolerable and mild. We think the high incidence of side effects in the control group may be due to stent-related symptoms and its complications and since this group did not receive any medication for reducing USRS, patients reported any minor symptoms. Perhaps in a large group study the result will be changed.

In a recent study by Mayor, reported high mortality rate for pregabalin and gabapentin suggested to use pregabalin with more caution (24). However, the patients of the present study did not report any severe side effects and the combination of pregabalin and solifenacin was well tolerated.

On the other hand, in our study, either pregabalin or solifenacin provided beneficial effects for improving USRSs. But the combination therapy of these two medications appear to respond better by reducing the disadvantages of each drug alone.

## Limitations

The method of data collection was a limitation in this study. USSQ questionnaire for each patient was filled according to the patient's statements, so self-reported data can contain several potential sources of bias that can affect the outcome of the study.

Given the limited number of cases in this study and carrying out in a single center, further multicenter trials with larger sample size, long-term follow-ups, and different methods are required to prove the research findings and effects of drugs in reducing USRS after DJ insertion in patients with ureteral stones.

## CONCLUSION

Combination therapy of pregabalin with solifenacin in patients with USRS has a significant effect on USSQ score with fewer bothersome side effects compared to either drug alone.

## APPENDIX 1 USSQ-EN JJ Stent in place.

### USSQ-EN JJ Stent in place

We are interested in various aspects of your health, as a result of the introduction of the stent (double J stent or probe DD) and its effect on your health.

Thank you fill out the questionnaire below, which presents different sections.

Thank you to answer all questions in each section. Only one answer per question.

Thank you to complete:

Today's Date: .. /.. /....

Birthday (Birth Date): .. /.. /....

#### I/ URINARY SYMPTOMS

Thank you to answer questions on urinary symptoms you felt as a result of the introduction of the J stent. Thank you to tick one box for each question.

##### U1. During the day, how often do you urinate, on average?

Every 4 hours or more □ 1

Every 3 hours □ 2

Every 2 hours □ 3

Every hour □ 4

Several times per hour □ 5

##### U2. During the night, how many times do you get up to urinate on average?

No □ 1

1 time □ 2

2 times □ 3

3 times □ 4

4 times or more 5 □

##### U3. Do you need to rush to the bathroom to urinate?

Never □ 1

Rarely (Less than a third of the time) □ 2

Sometimes (Between one and two thirds of the time) □ 3

Most of the time (over two-thirds of the time) □ 4

All the time □ 5

##### U4. Do you leak urine before you go to the toilet?

Never □ 1

Rarely (Less than a third of the time) □ 2

Sometimes (Between one and two thirds of the time) □ 3

Most of the time (over two-thirds of the time) □ 4

All the time □ 5

##### U5. Do you have urine leakage without feeling the need to urinate?

Never □ 1

Rarely (Less than a third of the time) □ 2

Sometimes (Between one and two thirds of the time) □ 3

Most of the time (over two-thirds of the time) □ 4

All the time □ 5

##### U6. How often have you had the feeling that your bladder is not emptying properly after urinating?

Never □ 1

Rarely (Less than a third of the time) □ 2

Sometimes (Between one and two thirds of the time) □ 3

Most of the time (over two-thirds of the time) □ 4

All the time □ 5

## APPENDIX 2 Multiple Comparisons.

**Table:**

Tukey HSD					
Dependent Variable	(I) Group	(J) Group	Mean Difference (I-J)	Std. Error	Sig.
Urinary Symptoms Score week 2	Pregabalin	Solifinacin	2.422	0.640	0.001
		Pregabalin + Solifinacin	4.672	0.640	0.000
		Control	1.766	0.640	0.031
	Solifinacin	Pregabalin + Solifinacin	2.250	0.640	0.003
		Control	−.656	0.640	0.735
	Pregabalin + Solifinacin	Control	−2.906	0.640	0.000
Urinary Symptoms Score week 4	Pregabalin	Solifinacin	2.563	0.486	0.000
		Pregabalin + Solifinacin	5.063	0.486	0.000
		Control	1.156	0.486	0.084
	Solifinacin	Pregabalin + Solifinacin	2.500	0.486	0.000
		Control	−1.406	0.486	0.021
	Pregabalin + Solifinacin	Control	−3.906	0.486	0.000
Pain Score week 2	Pregabalin	Solifinacin	−6.844	0.509	0.000
		Pregabalin + Solifinacin	4.328	0.509	0.000
		Control	−7.109	0.509	0.000
	Solifinacin	Pregabalin + Solifinacin	11.172	0.509	0.000
		Control	−.266	0.509	0.954
	Pregabalin + Solifinacin	Control	−11.438	0.509	0.000
Pain Score week 4	Pregabalin	Solifinacin	−6.601	0.368	0.000
		Pregabalin + Solifinacin	−.516	0.381	0.530
		Control	−8.266	0.381	0.000
	Solifinacin	Pregabalin + Solifinacin	6.085	0.368	0.000
		Control	−1.665	0.368	0.000
	Pregabalin + Solifinacin	Control	−7.750	0.381	0.000
General Condition Score week 2	Pregabalin	Solifinacin	−2.609	0.322	0.000
		Pregabalin + Solifinacin	−.313	0.322	0.767
		Control	−3.984	0.322	0.000
	Solifinacin	Pregabalin + Solifinacin	2.297	0.322	0.000
		Control	−1.375	0.322	0.000
	Pregabalin + Solifinacin	Control	−3.672	0.322	0.000
General Condition Score week 4	Pregabalin	Solifinacin	−1.719	0.247	0.000
		Pregabalin + Solifinacin	.500	0.247	0.181
		Control	−.625	0.247	0.057
	Solifinacin	Pregabalin + Solifinacin	2.219	0.247	0.000
		Control	1.094	0.247	0.000
	Pregabalin + Solifinacin	Control	−1.125	0.247	0.000
Work Performance Score week 2	Pregabalin	Solifinacin	.875	0.364	0.079
		Pregabalin + Solifinacin	3.000	0.364	0.000
		Control	−2.578	0.364	0.000
	Solifinacin	Pregabalin + Solifinacin	2.125	0.364	0.000
		Control	−3.453	0.364	0.000
	Pregabalin + Solifinacin	Control	−5.578	0.364	0.000
Work Performance Score week 4	Pregabalin	Solifinacin	−.516	0.380	0.528
		Pregabalin + Solifinacin	2.016	0.380	0.000
		Control	−.906	0.380	0.083
	Solifinacin	Pregabalin + Solifinacin	2.531	0.380	0.000
		Control	−.391	0.380	0.733
	Pregabalin + Solifinacin	Control	−2.922	0.380	0.000
Sexual Matters Score week 2	Pregabalin	Solifinacin	−1.969	0.189	0.000
		Pregabalin + Solifinacin	.156	0.189	0.841
		Control	−1.516	0.189	0.000
	Solifinacin	Pregabalin + Solifinacin	2.125	0.189	0.000
		Control	.453	0.189	0.080
	Pregabalin + Solifinacin	Control	−1.672	0.189	0.000
Sexual Matters Score week 4	Pregabalin	Solifinacin	−.438	0.118	0.001
		Pregabalin + Solifinacin	.297	0.118	0.059
		Control	−1.344	0.118	0.000
	Solifinacin	Pregabalin + Solifinacin	.734	0.118	0.000
		Control	−.906	0.118	0.000
	Pregabalin + Solifinacin	Control	−1.641	0.118	0.000
Additional Problems Score week 2	Pregabalin	Solifinacin	−3.578	0.188	0.000
		Pregabalin + Solifinacin	−.953	0.188	0.000
		Control	−1.578	0.188	0.000
	Solifinacin	Pregabalin + Solifinacin	2.625	0.188	0.000
		Control	2.000	0.188	0.000
	Pregabalin + Solifinacin	Control	−.625	0.188	0.006
Additional Problems Score week 4	Pregabalin	Solifinacin	−2.031	0.155	0.000
		Pregabalin + Solifinacin	−.141	0.155	0.800
		Control	−1.516	0.155	0.000
	Solifinacin	Pregabalin + Solifinacin	1.891	0.155	0.000
		Control	.516	0.155	0.005
	Pregabalin + Solifinacin	Control	−1.375	0.155	0.000
Dunnett t (2-sided)ᵃ					
Dependent Variable	(I) Group	(J) Group	Mean Difference (I-J)	Std. Error	Sig.
Urinary Symptoms Score week 2	Pregabalin	Control	1.76563*	0.64001	0.017
	Solifinacin	Control	−.65625	0.64001	0.607
	Pregabalin + Solifinacin	Control	−2.90625*	0.64001	0.000
Urinary Symptoms Score week 4	Pregabalin	Control	1.15625*	0.48594	0.048
	Solifinacin	Control	−1.40625*	0.48594	0.012
	Pregabalin + Solifinacin	Control	−3.90625*	0.48594	0.000
Pain Score week 2	Pregabalin	Control	−7.10938*	0.50919	0.000
	Solifinacin	Control	−.26563	0.50919	0.916
	Pregabalin + Solifinacin	Control	−11.43750*	0.50919	0.000
Pain Score week 4	Pregabalin	Control	−8.26563*	0.38123	0.000
	Solifinacin	Control	−1.66470*	0.36813	0.000
	Pregabalin + Solifinacin	Control	−7.75000*	0.38123	0.000
General Condition Score week 2	Pregabalin	Control	−3.98438*	0.32236	0.000
	Solifinacin	Control	−1.37500*	0.32236	0.000
	Pregabalin + Solifinacin	Control	−3.67188*	0.32236	0.000
General Condition Score week 4	Pregabalin	Control	−.62500*	0.24680	0.032
	Solifinacin	Control	1.09375*	0.24680	0.000
	Pregabalin + Solifinacin	Control	−1.12500*	0.24680	0.000
Work Performance Score week 2	Pregabalin	Control	−2.57813*	0.36426	0.000
	Solifinacin	Control	−3.45313*	0.36426	0.000
	Pregabalin + Solifinacin	Control	−5.57813*	0.36426	0.000
Work Performance Score week 4	Pregabalin	Control	−.90625*	0.38016	0.047
	Solifinacin	Control	−.39063	0.38016	0.606
	Pregabalin + Solifinacin	Control	−2.92188*	0.38016	0.000
Sexual Matters Score week 2	Pregabalin	Control	−1.51563*	0.18882	0.000
	Solifinacin	Control	.45313*	0.18882	0.046
	Pregabalin + Solifinacin	Control	−1.67188*	0.18882	0.000
Sexual Matters Score week 4	Pregabalin	Control	−1.34375*	0.11776	0.000
	Solifinacin	Control	−.90625*	0.11776	0.000
	Pregabalin + Solifinacin	Control	−1.64063*	0.11776	0.000
Additional Problems Score week 2	Pregabalin	Control	−1.57813*	0.18780	0.000
	Solifinacin	Control	2.00000*	0.18780	0.000
	Pregabalin + Solifinacin	Control	−.62500*	0.18780	0.003
Additional Problems Score week 4	Pregabalin	Control	−1.51563*	0.15479	0.000
	Solifinacin	Control	.51563*	0.15479	0.003
	Pregabalin + Solifinacin	Control	−1.37500*	0.15479	0.000

* = The mean difference is significant at the 0.05 level.

**A** = Dunnett t-tests treat one group as a control, and compare all other groups against it.

**Sig**. = Significant
